# Factors Positively Correlated with Hepatitis B Surface Antigen Seroconversion in Chronic Hepatitis B

**DOI:** 10.3390/jpm14040390

**Published:** 2024-04-05

**Authors:** Matthias Buechter, Arne Maria Günther, Paul Manka, Guido Gerken, Alisan Kahraman

**Affiliations:** 1 Department of Gastroenterology and Hepatology, University Clinic of Essen, University of Duisburg-Essen, 45147 Essen, Germany; arne1604@web.de (A.M.G.); guido@clausgerken.de (G.G.); alisan.kahraman@max-grundig-klinik.de (A.K.); 2 Department of Gastroenterology and Hepatology, Elisabeth Hospital, 58638 Iserlohn, Germany; 3 Department of Internal Medicine, University Hospital Knappschaftskrankenhaus, Ruhr-University Bochum, 44801 Bochum, Germany; paul.manka@kk-bochum.de; 4 Department of Gastroenterology and Hepatology, Helios Clinic, 42549 Velbert, Germany; 5 Department of Gastroenterology and Hepatology, Max Grundig Clinic, 77815 Bühl, Germany

**Keywords:** chronic hepatitis b, HBsAg seroconversion, seroclearance, hepatocellular carcinoma

## Abstract

Background and Aims: Chronic hepatitis B virus (HBV) infection is a global public health challenge since more than 250 million individuals are affected worldwide. Since different treatment modalities are available and not all patients are candidates for antiviral treatment, biomarkers that potentially predict the possibility of HBsAg clearance and seroconversion may be useful in clinical practice. Patients and methods: In this retrospective study, we aimed to identify factors positively correlated with HBsAg seroconversion in a large cohort of 371 chronic hepatitis B patients treated at a German tertial center between 2005 and 2020. Results: Seroconversion occurred in 25/371 (6.7%) and HBsAg loss in 29/371 patients (7.8%) with chronic HBV infection. Antiviral therapy was associated with a lower chance of seroconversion (seroconversion antiviral therapy 14/260 (5.4%) vs. therapy-naïve patients 11/111 (9.9%), *p* = 0.027). Seroconversion rates were higher in patients with (very) low titers of HBV DNA (best cut-off value 357 IU/mL) and quantitative HBsAg. The best cut-off value with regard to seroconversion was 357 IU/mL for HBV DNA (AUC 0.693 (95%-CI 0.063–0.422), sensitivity 0.714, specificity 0.729; *p* < 0.0005) and 33,55 IU/mL for HBsAg (AUC 0.794 (95%-CI 0.651–0.937), sensitivity 0.714, specificity 0.949; *p* < 0.0005). However, male gender was positively associated with seroconversion (seroconversion: males 7.6% vs. females 2.7%, *p* = 0.036). Conclusions: Treatment-naïve male chronic HBV patients with low viral load and inflammatory activity have the best chance to achieve seroconversion. In the absence of cirrhosis, antiviral therapy should therefore not be performed in this patient collective.

## 1. Introduction

Chronic hepatitis B virus (HBV) infection is a global public health challenge since more than 250 million individuals are affected worldwide. Approximately 45% of the world population lives in areas of high endemicity, including many African and Asian countries. Patients who are infected with HBV have an enhanced risk of complications such as the development of liver cirrhosis, liver decompensation and hepatocellular carcinoma (HCC), increasing morbidity and mortality dramatically. Although the majority of patients do not develop complications, it is estimated that during their lifetimes, 15–40% may develop serious sequelae of infection [1,2,3,4].

After exposure to HBV infection, proper management is necessary. With clinical outcomes from inactive carrier state to end-stage liver disease, close monitoring with periodic evaluation is required to prevent future complications.

Hepatitis B surface antigen (HBsAg) loss (seroclearance) or seroconversion is the ideal endpoint of antiviral therapy since this state implies functional cure. A negativation of HBsAg during the course of HBV infection usually confers an excellent long-term prognosis in the absence of cirrhosis or concurrent infections with hepatitis C or D. Thus, different treatment strategies have been explored to improve patients’ outcomes [5,6]. Nucleos(t)ide analogs (NAs), which suppress HBV DNA replication via inhibiting the reverse transcription of pregenomic RNA into HBV DNA, are considered the first-line treatment option. However, NAs fail to clear the HBV covalently closed circular DNA (cccDNA) and RNA replication intermediates. Thus, long-term treatment is needed for sustained viral suppression in chronic HBV patients. Although the new NAs are supposed to be drugs with a high genetic barrier to resistance and therefore reliably facilitate sustained viral suppression, functional cure (e.g., HBsAg loss or seroconversion) is rare due to the long-term persistence of viral DNA in the hepatocytes of treated patients [7,8,9]. The other cornerstone in hepatitis B treatment is interferon and its pegylated form, pegylated interferon α (PEG IFN α). PEG IFN α consists of a group of cytokines with antiviral activity; it mainly acts as an immunomodulator and enhances the host cell-mediated immune response. Although PEG IFN α has restricted efficacy in only a proportion of patients, responders to therapy may maintain a virologic response after drug withdrawal, and permanent HBeAg or even HBsAg clearance can be achieved. In contrast to NAs, the finite treatment duration (e.g., 6–12 months) offers an advantage to patients. However, its efficacy with regard to functional cure is still not satisfactory (seroclearance rates < 10%). In addition, multiple side-effects limit the use of PEG IFN α and lead to the frequent discontinuation of therapy in a substantial proportion of patients [10,11,12].

Both radiologic and serum biomarkers may yield valuable information on how to assess clinical disease evolution and associate available therapies. Viral markers such as HBV DNA, HBV RNA, quantitative HBsAg, and HBeAg are currently used to predict therapeutic response for chronic HBV patients undergoing NA therapy [5,7,9]. However, since different treatment modalities are available and not all patients are candidates for antiviral treatment, biomarkers that potentially predict the possibility of HBsAg clearance and seroconversion may be useful in clinical practice. In this retrospective study, we aimed to identify factors positively correlated with HBsAg seroconversion in a large cohort of chronic hepatitis B patients treated at a German tertial center between 2005 and 2020.

## 2. Patients and Methods

### 2.1. Patient Information and Ethical Considerations

In this retrospective study between 06/2005 and 02/2020, a total of 371 consecutive patients with chronic HBV infection were included. Chronic hepatitis B was defined as the persistence of hepatitis b surface antigen (HBsAg) for 6 months or more after acute infection with hepatitis B virus [13,14]. Study inclusion therefore necessitated two positive HBsAg results at an interval of at least 6 months. The second positive HBsAg verification was defined as the baseline date for study inclusion, data collection and follow-up. Consequently, patients without evidence for HBsAg persistence > 6 months were excluded from the study. The University Clinic of Essen ethics committee approved the retrospective, anonymous analysis of the data (17-7655-BO), and the study was conducted according to the principles expressed in the Declaration of Helsinki. All patients gave their written informed consent prior to study inclusion.

### 2.2. Data Collection, Laboratory Parameters, Antiviral Therapy and Definition of Seroconversion

The following epidemiological and laboratory parameters were recorded: age, body mass index (BMI, kg/m^2^), existence or occurrence of hepatocellular carcinoma (HCC), HBV-DNA (IU/mL), HBV genotype, HBsAg (IU/mL), anti-HBs (IU/mL), HBeAg (IU/mL), anti-HBe, gamma-GT (U/L), aspartate aminotransferase (AST; U/L), alanine aminotransferase (ALT; U/L), total bilirubin (mg/dL), cholesterol (mg/dL), high-density lipoprotein (HDL; mg/dL), low-density lipoprotein (LDL; mg/dL), triglyceride (mg/dL), plasma glucose (mg/dL) and glycated hemoglobin (HbA1c; %).

Structural changes in the liver parenchyma (fibrosis and steatosis) were assessed by transient elastography (TE). The measurement of liver stiffness (LSM, kPa) and controlled attenuation parameter (CAP, dB/m) was performed by Fibroscan (Echosens, Paris, France) according to the manufacturer’s recommendations. TE was performed with the patient in the supine position with the right arm in maximal abduction. The TE probe was used via the intercostal spaces with the probe on the right lobe of the liver. Each set of examinations was assisted by B-mode ultrasound (APLIO 500, Toshiba, Tokio, Japan) for the identification of a feasible probe position and the exclusion of perihepatic ascites. The median of at least 10 LSM values expressed in kPa was used as the representative measurement. The success rate was calculated as the number of valid measurements divided by the number of total measurements. According to the manufacturer’s recommendations, only patients with an interquartile range (IQR) < 30% of the median value and a success rate >60% were included in the analysis. According to the actual literature, significant fibrosis and liver cirrhosis were defined as LSM > 8 kPa and > 15 kPa, respectively. In addition, a CAP value > 222 dB/m was defined as significant steatosis [15,16,17,18,19,20,21,22,23,24].

Histology was obtained either percutaneously or via mini-laparoscopy-guided liver biopsy in some patients with, for example, overlap with metabolic dysfunction-associated steatotic liver disease (MASLD; diabetics with obesity) or possible overlap with autoimmune hepatitis, since hepatitis B sometimes triggers autoimmune phenomena (LKM positivity). However, liver biopsy was not routinely performed in our department.

Antiviral treatment was performed according to the German guideline recommendations (“Deutsche Gesellschaft für Gastroenterologie, Verdauungs- und Stoffwechselkrankheiten”; DGVS) and required HBV DNA > 2000 IU/mL, elevated ALT and/or at least moderate histological lesions, while all cirrhotic patients with detectable HBV DNA were treated. Antiviral therapy was performed by the use of either nucleos(t)ide analog (NA) to suppress HBV replication to induce the stabilization of HBV-induced liver disease and to prevent disease progression and HCC development or pegylated interferon-α to induce long-term immunological control with a finite duration treatment. However, drug treatment selection was based on individual parameters such as the severity of liver disease, pretreatment, co-morbidities, response probability, patients’ will, contraindications and local expertise according to published DGVS guidelines. Besides interferon-α, the following NAs were used: lamivudine, telbivudine, adefovir, entecavir, and tenofovir. First-line treatment was usually performed with drugs with good potency and high genetic barriers to drug treatment (e.g., tenofovir, entecavir). Patient monitoring followed DGVS recommendations for chronic HBV infection (determination of ALT, HBV DNA, HBeAg (if initially positive), HBsAg; sonography for HCC screening every 6 months). The general goal of antiviral HBV drug treatment was to suppress HBV DNA (below the detectable limit) and normalize ALT. In the case of an insufficient response, resistant variants were tested. Seroconversion was defined as the loss of HBsAg and the development of anti-HBsAg antibodies during follow-up [13,14,25,26].

### 2.3. Statistical Analysis

Statistical analysis was performed using SPSS (IBM, Chicago, IL, USA). For descriptive statistics, medians and IQRs were determined. All variables were tested for normal distribution with the Kolmogorov–Smirnov test, the Shapiro–Wilk test, and calculation of skew and kurtosis. The Mann–Whitney U test was used to compare differences between independent groups. Categorical data were tested with the chi-square test, and the Kruskal–Wallis test was used for multiple comparisons. To adjust for several risk factors, the multivariate logistic analysis was performed with all the variables found significant in univariate analysis in a single step. A *p* value < 0.05 was considered statistically significant.

## 3. Results

### 3.1. Demographic and Laboratory Data

Between 2005 and 2020, a total of 371 patients with chronic HBV infection and a well-documented long-term course were included in this retrospective study. The majority of the study population was male (*n* = 224, 60.4%), the mean age at initial diagnosis 48.08 ± 13.41 [23–84] years and the mean observation period 175.94 months. In addition, 10/371 patients (2.7%) were co-infected with hepatitis delta virus. Laboratory data on initial diagnosis are presented in Table 1, and the frequency of HBV genotypes is presented in Table 2.

### 3.2. HCC Incidence, Liver Elastography and CAP

Liver stiffness measurement (LSM) was available in 337/371 patients (90.8%). In total, 37/337 patients (11.0%) had liver cirrhosis (LSM > 15 kPa) and 85/337 patients (25.2%) had significant fibrosis (LSM > 8 kPa). CAP measurement was available in 256/371 patients (69.0%). Significant steatosis (CAP > 222 dB/m) was present in 103/256 patients (40.2%). Hepatocellular carcinoma (HCC) occurred in 8/371 cases (2.2%). LSM was available in six out of eight patients (cirrhosis: n = 1 (LSM 32.0 kPa), significant fibrosis n = 2 (LSM 11.6 kPa and 13.4 kPa), below cut-off n = 3 (LSM 4.4 kPa, 6.6 kPa, and 7.7 kPa)).

### 3.3. Antiviral Therapy

Antiviral therapy was performed in 260/371 patients (70.1%). In 117 out of 260 patients (45.0%), more than one antiviral substance was administered. A total of 58 patients (15.6%) received pegylated interferon for a mean timespan of 12.6 ± 8.6 months. Seroconversion was achieved in 4/58 of these patients (6.9%). Oral antiviral therapy was conducted by the use of tenofovir (n = 165), entecavir (n = 93), lamivudine (n = 63), adefovir (n = 55) and telbivudine (n = 29). Seroconversion was observed in 3.8% of patients (10/260) receiving oral antiviral therapies.

### 3.4. Factors Associated with Seroconversion

Seroconversion (the development of anti-HB antibodies) occurred in 25/371 (6.7%) and HBsAg loss in 29/371 patients (7.8%) with chronic HBV infection. The mean timespan from first diagnosis to seroconversion was 146 [7–478] months. The age at first diagnosis was not associated with seroconversion. However, male gender was positively associated with seroconversion (seroconversion: males 7.6% vs. females 2.7%, *p* = 0.036).

#### 3.4.1. Antiviral Therapy

Antiviral therapy was associated with a lower chance of seroconversion (seroconversion antiviral therapy 14/260 (5.4%) vs. therapy-naïve patients 11/111 (9.9%), *p* = 0.027). Although the rate of seroconversion was higher in the PEG-IFN (n = 4/58, 6.9%) group than the oral therapy group (n = 10/206, 4.9%), this difference did not reach statistical significance (*p* > 0.05). Data regarding seroconversion rates according to the presence or absence of antiviral therapy are presented in Table 3.

#### 3.4.2. HBV-Specific and Liver-Specific Laboratory Parameters and Liver Fibrosis Measured by Transient Elastography (TE)

**Figure 1:**
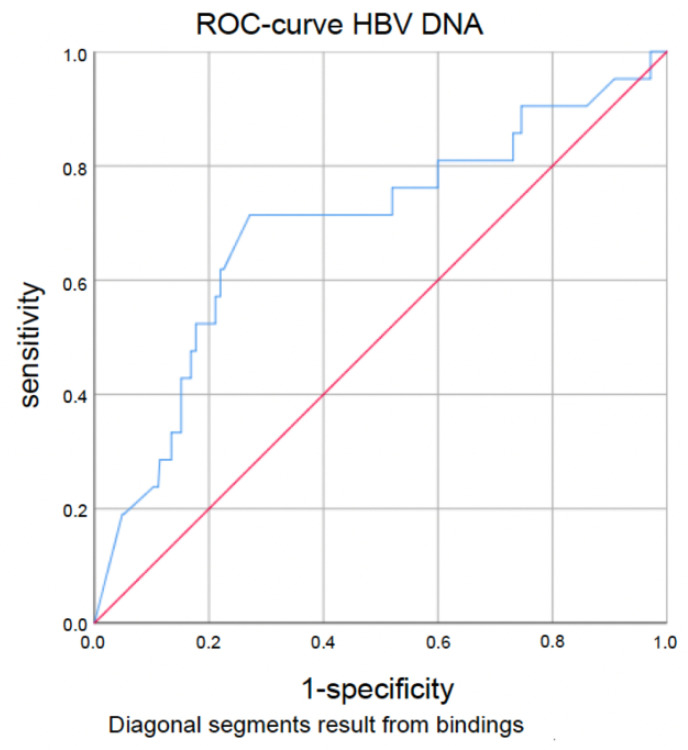
HBV DNA ROC curve according to seroconversion (cut-off 357 IU/mL, AUC 0.693 (95%-CI 0.063–0.422), sensitivity 0.714, specificity 0.729; *p* < 0.0005).

**Figure 2:**
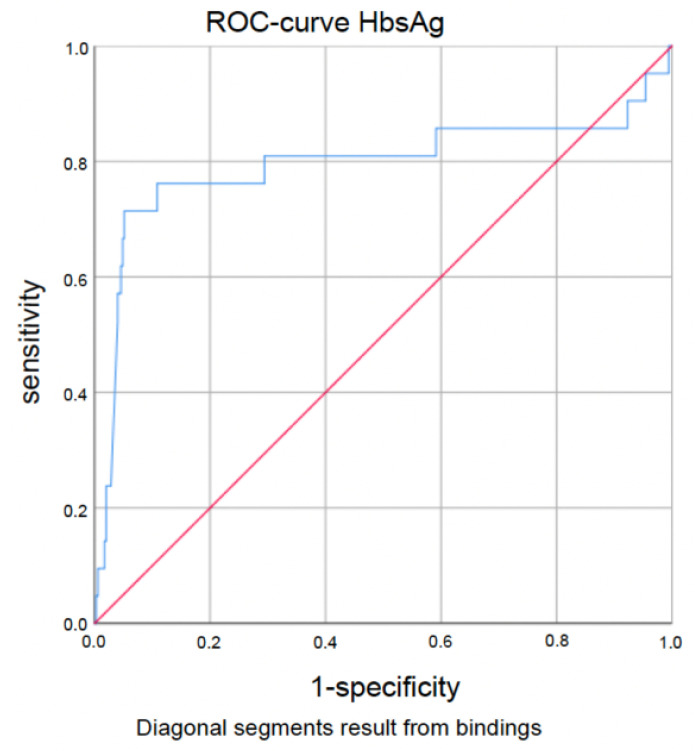
Quantitative HBsAg ROC curve according to seroconversion (cut-off 33.55 IU/mL, AUC 0.794 (95%-CI 0.651–0.937), sensitivity 0.714, specificity 0.949; *p* < 0.0005).

Seroconversion rates were higher in patients with (very) low titers of HBV DNA and quantitative HBsAg. The best cut-off value with regard to seroconversion was 357 IU/mL for HBV DNA (AUC 0.693 (95%-CI 0.063–0.422), sensitivity 0.714, specificity 0.729; *p* < 0.0005) and for HBsAg, 33.55 IU/mL (AUC 0.794 (95%-CI 0.651–0.937), sensitivity 0.714, specificity 0.949; *p* < 0.0005). Corresponding ROC curves are demonstrated in Figure 1 and Figure 2. However, the presence or absence of HBe antigen and anti-HBe antibodies was not a predictive factor regarding seroconversion.

Furthermore, γ-glutamyltransferase (GGT) was higher in patients attaining seroconversion. Statistical analysis, however, failed to reach significance, but showed a positive trend (mean GGT seroconversion 173.20 IU/mL vs. no seroconversion 44.89 IU/mL, *p* = 0.052). In addition, level of serum aspartat aminotransferase (AST), alanin aminotransferase (ALT) and bilirubin was not associated with seroconversion in chronic HBV patients.

**Figure 3:**
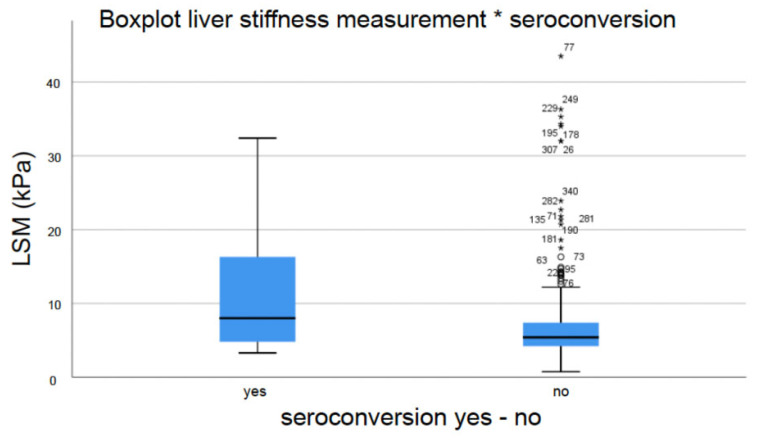
Correlation between liver stiffness measurement (LSM) and subsequent seroconversion (n = 337).

Interestingly, statistical analysis revealed a positive correlation between elevated liver stiffness and subsequent seroconversion (mean LSM seroconversion 8.2 ± 8.4 (3.3–32.4) kPa vs. mean LSM no seroconversion 5.4 ± 5.5 (3.2–43.5) kPa, *p* = 0.046) (Figure 3). When applying the previously cited cut-offs for significant fibrosis (>8 kPa) and cirrhosis (>15 kPa), subgroup analysis also showed a positive correlation (seroconversion significant fibrosis 11.0% vs. no significant fibrosis 3.4%, *p* = 0.008; seroconversion cirrhosis 16.2% vs. no cirrhosis 4.3%, *p* = 0.011). However, ROC analysis revealed an optimal LSM cut-off value with regard to seroconversion of 7.65 kPa (AUC 0.664, 95%-CI 0.528–0.800, sensitivity 0.579, specificity 0.7676, *p* = 0.016).

#### 3.4.3. Steatosis, Lipid Metabolism, Diabetes Mellitus and Obesity Were Not Associated with Seroconversion

Parameters associated with lipid metabolism disorders, metabolic dysfunction-associated steatotic liver disease (MASLD) and metabolic dysfunction-associated steatohepatitis (MASH) did not influence the probability of attaining seroconversion. The following parameters were analyzed: body mass index (BMI; seroconversion BMI > 25 kg/m^2^ 14/197 patients (7.11%) vs. seroconversion BMI < 25 kg/m^2^ 8/174 patients (4.60%), *p* = 0.20), concurrent diabetes mellitus/HbA1c > 5.7% (seroconversion 3/21 patients (14.29%) vs. no seroconversion 18/350 patients (5.14%), *p* = 0.095) and controlled attention parameter (CAP; CAP seroconversion 261,33 dB/m vs. CAP no seroconversion 240,51 dB/m, *p* = 0.218). Likewise, lipid metabolism parameters (serum cholesterol, triglycerides, high-density lipoprotein (HDL) and low-density lipoproteins (LDL)) had no influence on seroconversion (*p* > 0.05).

#### 3.4.4. Gender, GGT and Antiviral Therapy Were Independently Associated with Seroconversion

Multivariate logistic regression analysis was used to identify independent predictors of seroconversion. Significant parameters from the univariate analysis were included (antiviral therapy, gender, GGT, LSM, HBV-DNA, HBsAg). However, gender (OR: 0.233; CI: 0.060–0.898; *p* = 0.034), GGT (OR 0.993; CI: 0.990–0.997; *p* < 0.001) and antiviral therapy (OR: 3.97; CI: 1.302–11.722; *p* = 0.015) were independently associated with seroconversion.

## 4. Discussion

Functional cure in terms of HBsAg loss and the development of anti-HB antibodies (seroconversion) should be the ultimate goal in the treatment of patients with chronic hepatitis B with regard to drug withdrawal safety and improvements in prognosis. Although first-line drug therapy with NAs can suppress virus replication reliably (and thereby reduce the risk of complications such as the development of HCC and decompensation), lifelong therapy is usually required since the viral cccDNA persists in the nucleus of hepatocytes [27,28]. In selected patients, subcutaneous applied PEG IFN α represents a therapeutic alternative with the option of temporary limited treatment and the elimination of HBV. However, its use is often restricted by relevant side effects which leads to drug discontinuation in a significant proportion of patients [11,29,30,31,32].

Various algorithms regarding therapy indication in chronic HBV have been proposed, such as that by the American Association for the Study of the Liver Diseases (AASLD), the European Association for the Study of the Liver (EASL) or the Asian Pacific Association for the Study of the Liver (APASL). Although the recommendations slightly differ, antiviral treatment is usually indicated when chronic active hepatitis B disease is evident and at least one of the following criteria is fulfilled: (i) presence of advanced fibrosis or cirrhosis, (ii) HBV DNA level above at least 2000 IU/mL and (iii) persistently or repeatedly abnormal ALT/AST levels [13,14,26].

However, since different treatment modalities are available and not all patients are candidates for antiviral treatment, biomarkers that potentially predict the possibility of HBsAg clearance and seroconversion may be useful in clinical practice. In this retrospective study, we aimed to identify factors positively correlated with HBsAg seroconversion in a large cohort of 371 patients with chronic HBV infection treated at the University Hospital Essen between 2005 and 2020. In contrast to other studies, which were conducted in regions where HBV infection is endemic and usually acquired during the perinatal period or early infancy, Germany has low or intermediate patterns of endemicity. In population-based studies, approximately 0.3% of adults and 0.2% of infants were infected with HBV (HBsAg positive). In these studies, anti-HBc prevalence was 5.1% for adults and 0.5% for infants [1].

The overall HBsAg seroconversion rate in the mean observation time of approximately 176 months was relatively low, being 6.7% (25/371) of patients. However, these data match with data from the actual literature, in which short- and long-term HBsAg seroclearance and seroconversion rates are stated to be between 4.2% and 20.6% [33,34,35,36,37]. Interestingly, HBsAg seroconversion was significantly higher in therapy-naïve patients than in patients treated with antiviral drugs (11/111 patients (9.9%) vs. 14/260 patients (5.4%), *p* = 0.027). Subgroup analysis revealed that the seroconversion rate was higher in the PEG-IFN group (n = 4/58, 6.9%) than in the oral therapy group (n = 10/206, 4.9%), although this difference did not reach statistical significance (*p* > 0.05). However, antiviral therapy with NAs is very potent and usually leads to a rapid reduction in viral load and thereby in (systemic) inflammation. The consequently reduced confrontation/interaction between the virus and the host’s immune system might be an explanation for these findings with regard to pathogenesis.

Several studies have shown that serum HBV-DNA and quantitative HBsAg levels were the most significant indicators of seroclearance or seroconversion [34,38,39,40,41,42]. Similarly, in the present study, we observed that low HBV-DNA and quantitative HBsAg levels were associated with seroconversion, while the best cut-off values were 357 IU/mL and 34 IU/mL, respectively. Consequently, in selected patients with low HBsAg and HBV-DNA levels without cirrhosis, it might be useful to discontinue antiviral therapy with NAs to achieve spontaneous seroconversion. Fang et al. found that HBeAg-negative patients without cirrhosis and low HBsAg levels at the end of treatment who previously received entecavir or tenofovir had a high HBsAg loss rate after the discontinuation of treatment [43]. In addition, Choi and colleagues demonstrated that HBsAg loss occurred in 6.8% of HBeAg-negative HBV patients following NAs cessation [44]. Similarly, in the so-called “STOP-NUC trial”, a multicenter randomized-controlled trial, the research group of Thomas Berg and colleagues could demonstrate that stopping long-term NA treatment can induce a functional cure in HBeAg-negative patients with low HBsAg levels < 1000 IU/mL at the time point of NA treatment cessation [45]. The authors therefore conclude that finite therapy can be considered for chronic HBV patients on NA therapy. However, our data support the idea that a low viral load is an important factor for HBV cure and therapy decisions.

Some reports have demonstrated that males with chronic HBV infection seem to be more likely than females to experience seroclearance or seroconversion [34,39,46]. Likewise, male gender was associated with seroconversion in our cohort (seroconversion: males 7.6% vs. females 2.7%, *p* = 0.036).

Multivariate analysis showed statistically significant results with regard to gender (OR: 0.233; CI: 0.060–0.898; *p* = 0.034), GGT (OR 0.993; CI: 0.990–0.997; *p* < 0.001) and antiviral therapy (OR: 3.97; CI: 1.302–11.722; *p* = 0.015), which were independently associated with seroconversion.

Our data clearly indicate that treatment-naïve male chronic HBV patients with low viral load and inflammatory activity (elevated liver enzymes) should not receive antiviral therapy in the absence of liver cirrhosis, since they have a high chance of achieving spontaneous HBV seroclearance and seroconversion. As a future perspective, an increased understanding of trained immunity and how HBV establishes a permissive state in the host that bears no characteristics of immunologic tolerance may allow for the development of therapies targeted towards patients not currently indicated for treatment affecting both therapy initiation and termination [47].

Interestingly, a great proportion of individuals with chronic HBV infection showed signs of significant hepatic steatosis (CAP measurement > 222 dB/m; n = 103/256 (40.2%)) in terms of overlap with MASLD. Huang and colleagues investigated a great cohort of 4084 chronic HBV patients and found that in untreated HBeAg-negative patients, concurrent MASLD is associated with higher rates of seroclearance and seroconversion. Likewise, Li et al. retrospectively studied 6786 patients and identified that fatty liver was significantly and independently associated with a greater chance of achieving HBsAg seroclearance. The authors therefore conclude that metabolic dysfunctions have additive effects on the functional cure of chronic hepatitis B [48,49]. However, steatosis or related metabolic disorders were not associated with seroconversion in our cohort.

Chronic hepatitis B patients are at increased risk for hepatocellular carcinoma (HCC), especially when an advanced stage of liver fibrosis or cirrhosis is present. The risk for developing HCC increases from 6 to 37 times compared to control subjects. According to the actual literature, HCC incidence rates in chronic HBV subjects vary between 2 and 37% for overall incidence and 0.4 and 3% for annual incidence depending on the presence or absence of liver cirrhosis. There is solid evidence supporting the fact that, in addition to cirrhosis, the viral load reflected by HBV DNA and quantitative HBsAg levels is a strong risk factor for HCC development. Therefore, NA treatment can significantly reduce the incidence of HCC, though it does not completely eliminate the risk of HCC [50,51,52,53,54,55]. EASL and DGVS guidelines for HBV and HCC recommend HCC screening by abdominal ultrasound every six months with the optional determination of alpha-fetoprotein in chronic HBV patients with enhanced HCC risk (patients with advanced fibrosis or cirrhosis) [13,25]. Among our cohort, HCC development during a mean observation time of >175 months was rare (overall HCC incidence 2.2%) in chronic HBV patients, although a significant proportion had advanced fibrosis (25.2%) or cirrhosis (11.0%). The pathogenesis of HBV-induced HCC is thought to be multifactorial with both direct and indirect mechanisms. HBV-related HCC can also arise in non-cirrhotic livers, supporting the notion that HBV plays a direct role in liver transformation by triggering both common and etiology-specific oncogenic pathways in addition to stimulating the host immune response and driving liver chronic necroinflammation [2]. In any case, among our patient collective, 3/8 patients (37.5%) developed HCC without having cirrhosis. In our opinion, it is therefore reasonable to perform HCC screening in chronic HBV patients irrespective of whether fibrosis or cirrhosis is present.

Another interesting aspect of our study was the positive correlation between elevated liver stiffness and subsequent seroconversion (LSM seroconversion 8.2 ± 8.4 (3.3–32.4) kPa vs. LSM no seroconversion 5.4 ± 5.5 (3.2–43.5) kPa, *p* = 0.046 (Figure 3)), which applied to both significant fibrosis (seroconversion significant fibrosis 11.0% vs. no significant fibrosis 3.4%, *p* = 0.008) and cirrhosis (seroconversion cirrhosis 16.2% vs. no cirrhosis 4.3%, *p* = 0.011). The actual literature hardly offers any scientific data with regard to this consideration. Ming-Lun and colleagues, who investigated 81 patients dually infected with hepatitis B and C, found that baseline cirrhosis was a significant factor associated with HBsAg seroclearance [56]. However, the role of liver fibrosis in chronic HBV with regard to seroconversion still remains unknown.

We are aware of the limitations of our study, the most important of them being that it was a retrospectively performed single-center study.

In summary, we could demonstrate that treatment-naïve male chronic HBV patients with low viral loads and inflammatory activity (elevated liver enzymes) have the best chance of achieving seroconversion. The indication for antiviral therapy should therefore be provided carefully under these circumstances. When treated and surveilled at an expert center for liver diseases and according to the actual guidelines, HCC development seems to be relatively low though advanced fibrosis or cirrhosis is present.

## Figures and Tables

**Table 1:** Laboratory data on initial diagnosis.

Parameter	Value (Median; IQR)	Reference Value
HBV DNA [IU/mL]	3329 (356; 759626)	<10 IU/mL
HBsAg [IU/mL]	3823 (729; 12502)	<5 IU/mL
ꙋGT [U/L]	23 (14; 43)	<60 U/L
AST [U/L]	29 (22; 42)	<35 U/L
ALT [U/L]	37 (24; 65)	<45 U/L
bilirubin [mg/dL]	0.6 (0.5; 0.8)	0.1–1.2 mg/dL
cholesterol [mg/dL]	186 (161; 215)	<250 mg/dL
HDL [mg/dL]	50 (40; 63)	40–60 mg/dL
LDL [mg/dL]	114 (91; 139)	<150 mg/dL
triglycerides [mg/dL]	96 (71; 141)	<150 mg/dL
glucose [mg/dL]	88 (81; 95)	60–99 mg/dL
HbA1c [%]	5.5 (5.2; 5.8)	<5.7%

**Table 2:** Distribution of HBV genotypes among the study population.

Genotype	Frequency (n)	Proportion (%)
A	43	11.6
B	9	2.4
C	4	1.1
D	110	29.6
E	9	2.4
F	1	0.3
G	1	0.3
H	1	0.3
Unknown	193	52.0
Total	371	100.0

**Table 3:** Seroconversion rates according to presence or absence of antiviral therapy.

Therapy	Number of Patients Achieving Seroconversion	Number of Patients under That Specific Therapy	Fraction of Patients Achieving Seroconversion	Mean Timespan from Initial Diagnosis to Seroconversion in Months	Standard Deviation in Months
No therapy	11	111 (29.9%)	9.9%	167.5	148.2
Interferone	4	58 (15.6%)	6.9%	198	51.2
Telbivudine	2	29 (7.8%)	6.9%	93	48.1
Lamivudine	3	63 (17%)	4.8%	174.3	60.1
Any therapy (**NRTI+ *NNRTI+ Interferone)	10	260 (70.1%)	3.8%	152.7	78
Adefovir	2	55 (14.8%)	3.6%	140.5	19.1
**NRTI + *NNRTI	8	237 (63.9%)	3.3%	143.3	81.7
Entecavir	3	93 (25.1%)	3.2%	106.3	86.6
Tenofovir	4	165 (44.5%)	2.4%	146.3	77

*NNRTI, non-nucleoside reverse transcriptase inhibitor. **NRTI, nucleoside reverse transcriptase inhibitor.

## Data Availability

Data will be made available by the corresponding author upon reasonable request.

## Abbreviations

ALT: alanin aminotransferase; AST, aspartate aminotransferase; AUC, area under the curve; CAP, controlled attenuation parameter; ccc DNA, closed circular DNA; GGT, γ-glutamyltransferase; HbA1c, glycated hemoglobin; HBeAg, hepatitis B e-antigen; HBsAg, hepatitis B surface antigen; HBV, hepatitis B virus; HBV DNA, hepatitis B virus deoxyribonucleic acid; HBV RNA, hepatitis B virus ribonucleic acid; HCC, hepatocellular carcinoma; HDL, high-density lipoprotein; LDL, low-density lipoprotein; LSM, liver stiffness measurement; MASH, metabolic dysfunction-associated steatohepatitis; MASLD, metabolic dysfunction-associated steatotic liver disease; NA, nucleos(t)ide analog; PEG IFN α, pegylated interferon α; TE, transient elastography.
